# Evaluating Fouling Control and Energy Consumption in a Pilot-Scale, Low-Energy POREFLON Non-Aerated Membrane Bioreactor (LEP-N-MBR) System at Different Frequencies and Amplitudes

**DOI:** 10.3390/membranes12111085

**Published:** 2022-10-31

**Authors:** Runzhang Zuo, Yubin Yu, Canhui Song, Muxiang Liang, Xiejuan Lu, Dajun Ren, Xiaohui Wu, Feixiang Zan

**Affiliations:** 1 College of Resource and Environmental Engineering, Wuhan University of Science and Technology, Wuhan 430081, China; 2 School of Environmental Science and Engineering, Low-Carbon Water Environment Technology Center (HUST-SUKE), Huazhong University of Science and Technology, Wuhan 430074, China; 3 Key Laboratory of Water and Wastewater Treatment (HUST), MOHURD, Wuhan 430074, China; 4 Suke Environmental Protection Technology Co., Ltd., Suzhou 215026, China

**Keywords:** LEP-N-MBR, frequency, amplitude, nutrient removal, energy consumption

## Abstract

Continual aeration, a fouling control strategy that causes high energy consumption, is the major obstacle in the deployment of membrane bioreactors (MBRs) for wastewater treatment. In recent years, a technology has been developed which adopts mechanical reciprocity for membrane vibration, and it has been proven efficient for membrane scouring, as well as for saving energy: the low-energy POREFLON non-aerated membrane bioreactor (LEP-N-MBR). In this study, a pilot-scale LEP-N-MBR system was designed, established, and operated at various frequencies and amplitudes, and with various membrane models, so as to evaluate energy usage and membrane fouling. The results showed that a slower TMP rise occurred when the frequency and amplitude were set to 0.5 Hz and 10 cm, respectively. Under a suitable frequency and amplitude, the TMP increasing rate of model B (sealed only with epoxy resin) was slower than that of model A (sealed with a combination of polyurethane and epoxy resin). The average specific energy demand (SED) of the LEP-N-MBR was 0.18 kWh·m^−3^, much lower than the aerated MBR with 0.43 kWh·m^−3^ (obtained from a previous study), indicating a significant decrease of 59.54% in the SED. However, the uneven distribution of sludge within the membrane tank indicated that the poor hydraulic mixing in the reactor may result in sludge accumulation, which requires further operational optimization. The findings of this pilot-scale study suggest that the LEP-N-MBR system is promising and effective for municipal wastewater treatment with a much lower level of energy usage. More research is needed to further optimize the operation of the LEP-N-MBR for wide application.

## 1. Introduction

A rise in nitrogen- and phosphorus-containing compounds is caused by the discharge of untreated or inadequately treated municipal or industrial wastewater into aquatic systems, which may cause eutrophication issues [1]. The removal of these nutrients from wastewater has a significant influence on the environmental safety of the receiving water bodies. In comparison with conventional activated sludge (CAS) systems, membrane bioreactors (MBRs), which combine activated sludge systems with membrane filtration, are promising and effective for municipal wastewater treatment [2,3]. MBRs have several advantages, including the production of high-quality effluent, a small footprint, and low sludge-generation rates [4]. However, membrane-based filtration methods are eventually plagued by membrane fouling, which raises the transmembrane pressure (TMP) and decreases the operating flux [5]. It has been found that a full-scale MBR requires specific filtration energy in the range of 0.5–1 kWh·m^−3^, with the increase in the biofouling-induced TMP accounting for 30–70% of the total energy consumption [6]. Another significant maintenance cost is the periodic membrane cleaning necessitated by membrane fouling. In this context, many efforts towards fouling control have been devoted to reducing the energy consumption of MBRs. Aeration is one of the hydrodynamic techniques used to alleviate membrane fouling in submerged MBRs, which can provide local shear and turbulence near the membrane surface, reducing particle deposition on the membrane surface [7]. However, it is difficult for aeration to disperse the bubbles uniformly on the hollow fiber membrane [8]. Additionally, the shear forces produced by aeration are moderate due to hydrodynamic limits; hence, aeration must be optimized by considering operational factors, such as aeration rate, bubble size, and aeration mode [9]. On the other hand, energy consumption by aeration often accounts for 50–90% of the total energy consumption and more than 30% of the entire operating cost [10]. Therefore, it is necessary to explore alternatives for effectively alleviating membrane fouling while achieving a lower level of energy consumption.

Membrane vibration, a mechanical method that imposes shear on membrane surfaces, has recently been proposed and investigated as a means to minimize the energy consumption of the fouling control systems in MBRs [11]. Membrane vibration is the dynamic shear stress caused by the relative motion between the membrane surface and surrounding fluid to alleviate membrane fouling [12]. Mechanical systems offer better efficiency and cost-effectiveness than traditional control strategies. Furthermore, shear caused by vibration may be dispersed more uniformly throughout the membrane [13]. The efficiency of membrane vibration for fouling control is dependent on vibration frequency and amplitude. Different frequencies of vibration produce different shear rates on the membrane surface [14]. Ullah et al. found that membrane vibration could reduce pore-blocking with an increase in the membrane vibration frequency [15]. A higher vibration amplitude had a positive effect on both the feeding rate and the effluent quality [16]. However, shear forces are larger than Brownian, inertial, and drag forces, keeping particles away from the membrane surface and making pore-blocking the dominant phenomenon at higher vibration frequencies and amplitudes [17]. In addition, the effect of potting material on the alleviation of fouling by membrane vibration has not been studied. More importantly, increasing the scale of an MBR will significantly impact its performance. Thus, it is critical to evaluate the impacts of the vibration frequency, amplitude, and seal material on fouling performance and energy consumption on a pilot-scale MBR system, the practical conditions of which could be beneficial for its wide application.

The objective of this study was to evaluate the overall performance of a pilot-scale low-energy POREFLON non-aerated membrane bioreactor (LEP-N-MBR) system to verify its potential application for municipal wastewater, specifically in regard to energy consumption. A LEP-N-MBR system was designed, established, and operated under different operational conditions, i.e., vibration frequency, vibration amplitude, and potting material. Also, the potential application of the LEP-N-MBR system is discussed.

## 2. Materials and Methods

### 2.1. The Pilot-Scale LEP-N-MBR System

**Figure 1:**
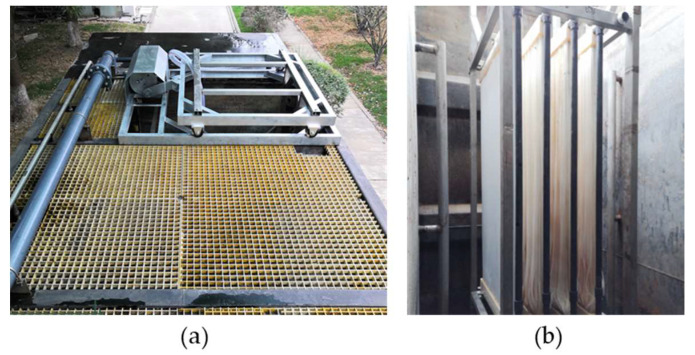
(**a**) The device for reciprocating motion in the pilot-scale LEP-N-MBR system; (**b**) The membrane module in the pilot-scale LEP-N-MBR system.

**Figure 2:**
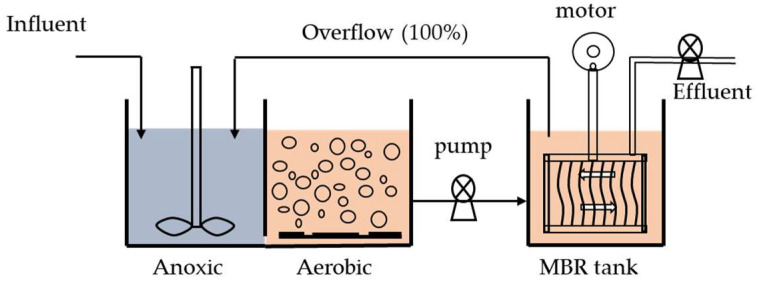
The schematic diagram of the pilot-scale LEP-N-MBR system.

The pilot-scale LEP-N-MBR consists of a biological treatment unit and a submerged, hollow fiber, vibrating membrane unit in Suzhou, Jiangsu (China), as shown in Figure 1. The process flow diagram for the LEP-N-MBR system is shown in Figure 2. The three biological reactors had operating volumes of 6.2 m^3^, 12.5 m^3^, and 6.2 m^3^ for the anoxic, aerobic, and membrane tanks, respectively. The hydraulic retention time (HRT) of each zone was 3 h for the anoxic, 6 h for the aerobic, and 3 h for the pilot system, respectively. The solid retention time (SRT) was 30 d, and the concentration of mixed liquor suspended solids (MLSS) was 8000–13000 mg·L^−1^. The processing capacity of the pilot-scale LEP-N-MBR was 100 m^3^ d^−1^.

The membrane material, polytetrafluoroethylene, was provided by Sumitomo Electric Industries (Japan), and the membrane modules were assembled by Suke Ltd. (China). The membrane modules were moved by a cam-driven reciprocating frame that was linked to the container. The external configuration mainly consisted of a motor and membrane modules. Wheels supported the reciprocating frame, which was reciprocated by the motions of a cam, which was, in turn, coupled to a motor. In order to find the optimal conditions, the pilot-scale LEP-N-MBR was primarily evaluated on the impact of amplitude (5, 7.5, and 10 cm) and frequency (0.5 and 0.6Hz) on the effluent quality and TMP during 130 days of operation. The operating flux was set up as 17–28 LMH. The membrane tank received feed water that had been emptied from an activated sludge tank. The operating conditions and properties of the vibrating membrane are shown in Table 1. In the pilot LEP-N-MBR, two membrane models were used: model A, which was sealed with polyurethane and epoxy resin, and model B, which was only sealed with epoxy resin. Both models were constructed with the same material, but they were encapsulated in different ways. In order to mimic the real conditions of a full-scale MBR plant, the filtering was carried out with a cycle of 10 min. The cycle of filtration included filtration and relaxation. The 10-min filtration cycles consisted of a 9-min filtration period and a 1-min rest period. Backwashing for 60 s was conducted every 60 min. Chemical cleaning was performed once per week with 1000 mg·L^−1^ of sodium hypochlorite, 1000 mg·L^−1^ of sodium hydroxide, and 2000 mg·L^−1^ of citric acid. The chemical cleaning involved a 30-min soaking time using NaOCl and NaOH, followed by a 60-min soaking time using citric acid, and finally a 10-min relaxing time with reciprocating motion. The reciprocating motor was continuously operated during the soaking period to improve the chemicals’ cleaning performance.

### 2.2. Sampling and Analytical Methods

The samples of influent and permeate were filtered using a sterile syringe filter at a 0.45 μm pore size before further measurement. The concentrations of chemical oxygen demand (COD), total nitrogen (TN), ammonia (NH_3_-N), total phosphorus (TP), MLSS, and SV30 were carried out according to the standard methods [18].

### 2.3. Specific Energy Demand Calculation

Specific energy demand (SED) can be expressed as Equation (1):

(1) $$\mathrm{SED}=\frac{P\;\times \;T}{V},$$

where SED is the specific energy demand (kWh·m^−3^); P is the power requirement (kW); T is the operating time (h); and V is the volume of permeate (m^3^). The SED of the aerated MBR was estimated as the energy consumption of the air blower at a determined rated power (0.75 kW) under long-term operation.

## 3. Results and Discussion

### 3.1. Frequency and Amplitude

**Figure 3:**
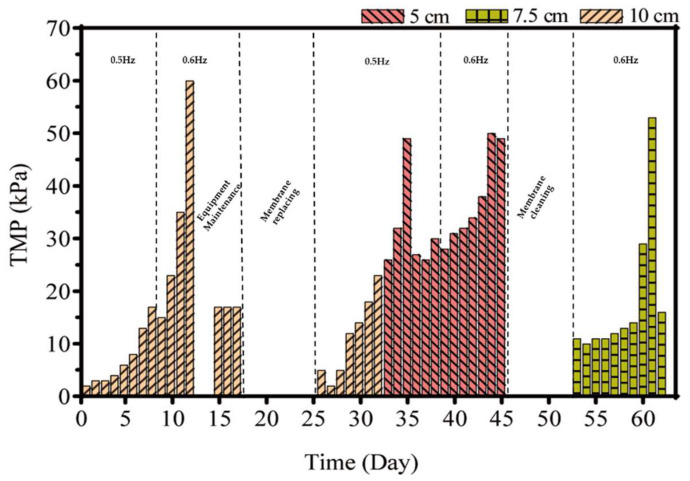
The profile of TMP under different frequencies (0.5 and 0.6 Hz) and amplitudes (5, 7.5, and 10 cm) in LEP-N-MBR.

The membrane vibrating frequency and amplitude are the main parameters of the design and operation in MBRs [19]. The pilot-scale LEP-N-MBR system was operated at different frequencies (0.5 and 0.6 Hz) and amplitudes (5, 7.5, and 10 cm) for about 65 days (Figure 3). With the operating amplitude maintained at 10 cm, the TMP gradually increased in the first 8 days at a frequency of 0.5 Hz, while a slight increase of frequency (0.6Hz) could significantly promote the TMP. The chemical cleaning was performed when the TMP was at 60 kPa. The equipment was maintained on days 13–14 due to the wash-out of the sludge. Thereafter, the membrane area was decreased from 100 m^2^ to 75 m^2^ to verify whether the system was stable on days 15–17. A damaged membrane frame and membrane module were replaced on days 18–25. As for the operating amplitude at 5 cm, the TMP rapidly increased from 26 kPa to 49 kPa on days 33–35, and chemical cleaning was performed on day 36. A lower operating amplitude may accelerate TMP at about 60 kPa, relatively higher than under other conditions. The alleviation of membrane fouling could benefit more with the frequency at 0.5 Hz. With the operating amplitude at 7.5 cm and the frequency at 0.6Hz, the TMP increased from 11 kPa to 60 kPa on days 53–61 and more quickly than that with amplitude set at 10 cm, and chemical cleaning was performed on day 62. The results showed that the suitable optimization amplitude and frequency were 10 cm and 0.5 Hz, respectively. Changes in TMP are highly dependent on membrane vibrating frequency and amplitude [20], and a high frequency and high amplitude may lower the increase in TMP. The different result observed in this study deserved further investigation.

### 3.2. Potting Material

**Figure 4:**
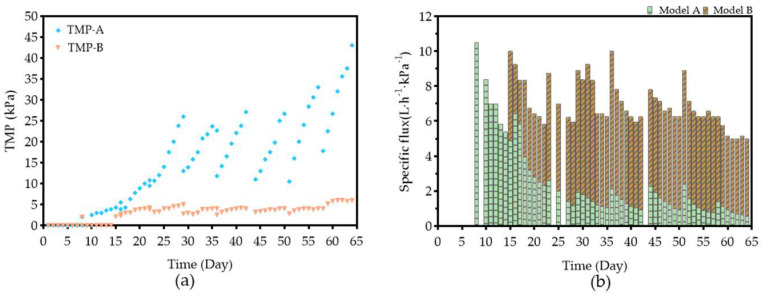
(**a**) The change in TMP with different membrane models in LEP-N-MBR. (**b**) The profile of specific flux under different membrane models in LEP-N-MBR.

Under constant flux conditions, membrane fouling may be manifested as an increase in TMP with increasing operation time. The TMP of the LEP-N-MBR across membrane models A and B exhibited the different trends, as shown in Figure 4a. The operating flux of models A and B was set at 17 LMH (days 1–7), 21 LMH (days 8–16), 25 LMH (days 17–22), 28 LMH (days 22–28), and 25 LMH (days 29–58), respectively. On days 59–65, the flux of model A maintained at 25 LMH, while the flux of model B increased to 30 LMH. Different materials used to seal membranes may cause various impacts on operational performance. In model A, it was clear that the TMP increased slowly under low operational flux in the early stages, and the TMP increased rapidly with increasing operating time. The membrane of model A was changed six times during the 65-day operation. On the other hand, the TMP in model B was maintained at a very low level in the early stage, and it started to rise slowly with the increase in operation time beginning on day 15. This suggests that model B, which was only sealed with epoxy resin, performed better than model A (sealed with a polyurethane and epoxy resin combination) in the LEP-N-MBR. Because of their remarkable adhesion and corrosion resistance, epoxy resins are the most extensively used flexible material for coating applications [21]. The different addition ratios of polyurethane have different results. Self-polymerization will occur and destroy the uniformity of the network structure of membrane models with high addition ratios of polyurethane [22]. In this study, the result showed that the addition of polyurethane may reduce the strength of the membrane module and increase the fouling rate by the shear generated by aeration.

The specific flux (SF), the proportion of flow to TMP, is a key factor for membrane fouling. Membrane fouling is severe when the SF is smaller [23]. In the first period, the SF of model A quickly reduced from 16.8 to 0.81 L h^−1^ kPa^−1^ (Figure 4b). The foulants immediately gathered on the model A membrane surface. SF could not recover to an optimal condition after the membrane was cleaned. In comparison with model A, the SF of model B gradually declined over the first period, from 13.3 to 4.25 L h^−1^ kPa^−1^, and recovered quickly after cleaning. Therefore, model B showed a better performance compared with model A in the pilot-scale LEP-N-MBR system.

### 3.3. Nutrient Removal

The performance of nutrient removal in the pilot-scale LEP-N-MBR system was further analyzed using an appropriate membrane model (model B), an optimized frequency (0.5 Hz), and an amplitude of 10 cm on day 130. The variations of COD, TP, TN, and NH_3_-N are presented in Table 2. Generally, the COD and NH_3_-N could be effectively removed in the LEP-N-MBR system, while TN and TP could not satisfy the discharging standard (A-class level in China: TN < 10 mg/L and TP < 0.5 mg/L). To be specific, the removal efficiency of the COD and NH_3_-N, with effluent concentrations of 27.25 mg/L and 0.30 mg/L, could reach about 88.22% and 98.79%, respectively. Although the influent COD concentration fluctuated variably, the COD removal capability in the LEP-N-MBR was quite stable. The system achieved a TN removal of 62.99% in the effluent. Because there was no aeration in the membrane tank of the LEP-N-MBR system and anoxic conditions were maintained, more endogenous denitrification may occur in the membrane tank, thus increasing the removed amount of TN [24]. Meanwhile, 45.16% of the initial phosphorus loading was measured in the effluent of the LEP-N-MBR after treatment. The low phosphorus removal efficiency of the LEP-N-MBR could be attributed to the absence of an anaerobic tank that allows for better phosphorus uptake of polyphosphate accumulating organisms [11]. These findings suggested that a vibrating MBR might be a potential alternative method for treating domestic wastewater on a pilot scale. Furthermore, the optimization of the LEP-N-MBR system is of significance to further improving the efficiency of nutrient removal. For instance, limited or intermittent aeration should be provided to increase the mixing condition for mass transfer, which can not only promote the sludge growth at a low level of dissolved oxygen, but it can also alleviate membrane fouling without high energy consumption. Therefore, more research is deemed necessary to investigate the integrated measures to improve the overall operation of the LEP-N-MBR.

### 3.4. Sludge Characteristics

**Figure 5:**
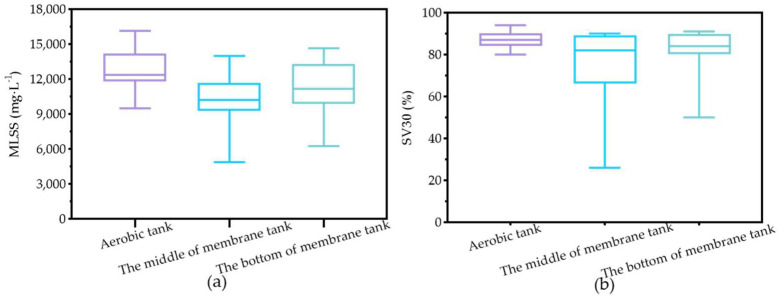
The MLSS (**a**) and SV_30_ (**b**) in the aeration tank and the middle and bottom of the membrane in the LEP-N-MBR system.

Figure 5 depicted the overall sludge characteristics in terms of MLSS and SV_30_. The concentration of MLSS was maintained between 4500 and 15,000 mg L^−1^ in the membrane tank. The concentration of MLSS in the middle, ranging from 4500 to 14,000 mg·L^−1^, is lower than that in the bottom, ranging from 6200 to 15,000 mg·L^−1^. The inhomogeneous distribution of MLSS may be attributed to a lack of aeration without enough biomass mixing in the membrane tank. The concentration of MLSS between 9500 and 16,000 mg·L^−1^ in the aerobic tank was higher than that in the membrane tank. This may be due to a lower agitation and a lower DO level, thus limiting the growth of sludge in the membrane tank. The SV_30_ of the aerobic tank was similar to that at the bottom of the membrane tank, while the SV_30_ of the middle of the membrane tank was lower. The even distribution of sludge in the membrane tank benefited the effect of the overflow to the anoxic tank and can further promote the efficiency of the nutrient removal. These results further showed that there was an uneven distribution of sludge in the membrane tank and further optimization may be needed.

### 3.5. Energy Consumption

**Figure 6:**
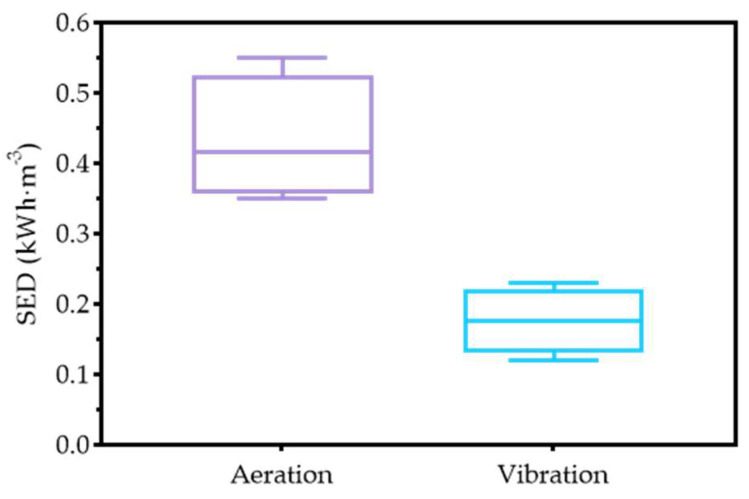
The different SEDs of aeration and vibration in an MBR.

SED is an essential indicator for evaluating the energy consumption of fouling control measures in an MBR. As shown in Figure 6, the average SED of the LEP-N-MBR, with 0.18 kWh·m^−3^, was much lower than that in the previous data for the aerated MBR, with 0.43 kWh·m^−3^. The SED of the aerated MBR was estimated with the energy consumption of the air blower at a determined rated power (0.75 kW) under long-term operation, which is similarly supported by previous studies [20,24]. Typical SED values for an aerated MBR are reported to be in the range of 0.5–0.7 kWh·m^−3^ [25]. The SED of the LEP-N-MBR was approximately 59.54% lower than that of the aerated MBR under similar operational conditions. These results demonstrated that vibrating membrane technology could significantly reduce energy consumption while controlling membrane fouling in an MBR, which has also been validated by other investigations [13,26]. Compared with the aerated MBR, the primary energy consumption in the LEP-N-MBR is caused by the motor rotation, which improves the shear stress along the membrane surface, instead of pumping air for membrane scouring and biomass mixing [20]. The results obtained from the pilot-scale study further confirmed that the LEP-N-MBR could be an energy-efficient option for effective fouling control in wastewater treatment.

### 3.6. Potential Application

This study has demonstrated that vibration in an MBR is effective in treating municipal wastewater with a better level of energy usage on a pilot scale. The operation of the LEP-N-MBR system was highly dependent on the membrane vibrating frequency, amplitude, and potting material, which could affect the membrane fouling and cleaning. Moreover, nutrient removal could be satisfactorily achieved for COD and ammonia, while that for TN and TP may require further optimization. More importantly, the energy consumption in the pilot-scale LEP-N-MBR system showed significant merit, as compared with an aeration-based MBR, with a reduction of 59.54%. This provides an opportunity for the wide application of the MBR process in areas where the economic impact is largely considered. However, this pilot-scale LEP-N-MBR system still needs further optimization in terms of nutrient removal, the vibrating module, and operating conditions. Also, a comprehensive environmental and economic analysis is deemed necessary so as to compare its energy and cost from a holistic aspect.

## 4. Conclusions

To evaluate the effect of fouling control under membrane vibration, this study comprehensively investigated nutrient removal and energy consumption under different frequencies and amplitudes in a pilot-scale LEP-N-MBR. The results showed that a slower TMP rise occurred when the frequency and amplitude were set to 0.5 Hz and 10 cm, respectively. Under the optimal frequency and amplitude, the TMP increasing rate of model B was slower than that of model A. Meanwhile, the average SED of the LEP-N-MBR significantly decreased the SED by 59.54%, as compared to that of the aerated MBR. However, the uneven distribution of sludge within the membrane tank indicated that poor hydraulic mixing in the reactor may result in sludge accumulation, which requires further operational optimization. The findings of this study suggest that the LEP-N-MBR system is promising and effective for municipal wastewater treatment with a much lower level of energy usage, while more research is needed to further optimize the operation for its wide application.

## Figures and Tables

**Table 1:** The membrane characteristics and operation conditions of the LEP-N-MBR system.

Parameters	Value/Setpoint
Material	Polytetrafluoroethylene (PTFE)
Pore size	0.1 μm
Membrane modules	4
Surface area per module	25 m^2^
Total membrane surface	100 m^2^
Operation flux	17–28 LMH
MLSS	8000–13,000 mg·L^−1^
Vibrating frequency	0.5 and 0.6 Hz
Vibrating amplitude	50, 75, and 100 mm
Filtration: idling	9 min: 1 min
Chemical cleaning	Once per week
HRT	12 h
SRT	30 d
Running time	130 d

**Table 2:** The concentrations of influent and effluent, and the removal efficiency of COD, TP, TN, and NH_3_-N in the LEP-N-MBR system.

Parameters	Influent (mg·L^−1^)	Effluent (mg·L^−1^)	Removal Efficiency (%)
**COD**	267.50 ± 124.85	27.25 ± 11.25	88.22 ± 6.54
**TN**	32.40 ± 8.68	12.46 ± 6.19	62.99 ± 11.06
**NH_3_-N**	25.51 ± 8.46	0.30 ± 0.15	98.79 ± 0.52
**TP**	3.90 ± 0.86	2.21 ± 0.51	45.16 ± 13.04

## Data Availability

Not applicable.
